# Found in Translation: The Utility of *C. elegans* Alpha-Synuclein Models of Parkinson’s Disease

**DOI:** 10.3390/brainsci9040073

**Published:** 2019-03-28

**Authors:** Anthony L. Gaeta, Kim A. Caldwell, Guy A. Caldwell

**Affiliations:** 1 Department of Biological Sciences, The University of Alabama, Tuscaloosa, AL 35487, USA; algaeta@crimson.ua.edu (A.L.G.); kcaldwel@ua.edu (K.A.C.); 2 Departments of Neurology and Neurobiology, Center for Neurodegeneration and Experimental Therapeutics, Nathan Shock Center of Excellence in the Basic Biology of Aging, University of Alabama at Birmingham School of Medicine, Birmingham, AL 35492, USA

**Keywords:** alpha-synuclein, Parkinson, *C. elegans*, neurodegeneration, dopamine

## Abstract

Parkinson’s Disease (PD) is the second-most common neurodegenerative disease in the world, yet the fundamental and underlying causes of the disease are largely unknown, and treatments remain sparse and impotent. Several biological systems have been employed to model the disease but the nematode roundworm *Caenorhabditis elegans (C. elegans)* shows unique promise among these to disinter the elusive factors that may prevent, halt, and/or reverse PD phenotypes. Some of the most salient of these *C. elegans* models of PD are those that position the misfolding-prone protein alpha-synuclein (α-syn), a hallmark pathological component of PD, as the primary target for scientific interrogation. By transgenic expression of human α-syn in different tissues, including dopamine neurons and muscle cells, the primary cellular phenotypes of PD in humans have been recapitulated in these *C. elegans* models and have already uncovered multifarious genetic factors and chemical compounds that attenuate dopaminergic neurodegeneration. This review describes the paramount discoveries obtained through the application of different α-syn models of PD in *C. elegans* and highlights their established utility and respective promise to successfully uncover new conserved genetic modifiers, functional mechanisms, therapeutic targets and molecular leads for PD with the potential to translate to humans.

## 1. Introduction

“I once was lost, for now I’m found”

-*Amazing Grace, John Newton* (1779)

Some of the most pervasive diseases that plague humanity include those that impair the nervous system, which are often slow to manifest and cause distress and suffering to those affected and the people dear to them. One such disease is Parkinson’s Disease (PD) and it accounts for the second highest number of cases of any neurodegenerative disease worldwide. PD is characterized by the degeneration and ultimate failure of the dopaminergic system in the brain, leading to the movement- and dementia-related symptoms for which it is known. Although the scientific community has made great strides in fundamentally understanding PD at the cellular and molecular level since its formal classification in the early 19th century, a cure for the disease remains elusive and treatment options are limited in both quantity and effectiveness. Several mutations in genes and corresponding proteins have been identified in people with familial forms of PD and scientists have characterized what roles these proteins have in cells [1,2,3,4]. These are referred to as *PARK* genes and mutations in them substantially increase susceptibility to acquiring PD in humans. In spite of this, there has not been a translation of this increase in knowledge into actual effective treatments for the disease. This highlights the need for more studies utilizing innovative animal models that recapitulate PD pathology. Taking this approach allows for the identification and characterization of PD related modifiers that have the potential to confer protective effects. Despite advances like organoid cultures and induced-pluripotent stem cell modeling, an intact living organism with dopamine (DA) neurons and a circuitry that regulates quantifiable behavioral responses in which various mutations or knockdown and/or overexpression of genes hypothesized to modify DA neurodegeneration can be investigated over time, provides distinct and complementary advantages in the investigation of PD modifiers.

There are several model organisms that can be used to model PD and ultimately increase the probability of discovering modifiers of DA neurodegeneration to accelerate subsequent development of translational treatments for humans. Each model organism has unique advantages and disadvantages. For instance, mammalian models offer the advantage of likeness, since humans have tissues and organs, as well as genetic and cellular similarities with other mammals. Mammalian PD models may yield results from studies that have a higher chance of directly translating to humans. However, mammalian models have the disadvantage of abundance—generally, mammals are relatively large and expensive to house and maintain, and because of this it is difficult to obtain large numbers to test. This can result in less effective screens and less statistical power when determining whether an effect of a treatment or condition is significant. 

On the opposite side of the model organism spectrum are very small and less complex animals like the nematode roundworm *Caenorhabditis elegans* (*C. elegans*). This invertebrate offers the opposite advantages and disadvantages in comparison to mammals. Due to their small size, high reproductive rate and low cost of maintenance, worms can provide a much greater abundance of individuals for testing. However, they have a disadvantage in likeness, since their anatomy and behaviors are, in general, vastly different from mammals and humans. However, it is important to note that this disadvantage is not as detrimental as it may seem. *C. elegans* shares over 80% genetic homology with humans in terms of genes that are linked to disease [5]. Despite the disparity between humans and worms, *C. elegans* also have DA neurons and provide researchers with great statistical power and confidence in experiments aimed at the discovery and characterization of genes, mechanisms and other factors that are involved in the modification of DA neuron degeneration that would otherwise not be possible in more complex organisms like mammals. By identifying and functionally evaluating modifiers of dopamine-associated phenotypes in *C. elegans* and then validating their activity in more complex model organisms like mammals, an efficient “assembly-line” could create a fast-track to the creation of treatments for PD in humans. This approach using *C. elegans* has been put into practice and has culminated in studies that have furthered progress to this end [6,7,8,9]. Taking all of this into account, the need for more studies utilizing powerful model systems like *C. elegans* for PD research has never been more critical. Along with all of the recently developed and upcoming biotechnologies that allow for the ease of sequencing and the generation of specific mutations, such as Next Generation Sequencing (NGS) and CRISPR-Cas9, a greater understanding of PD pathology and the discovery of effective treatments are closer than ever. 

**Figure 1:**
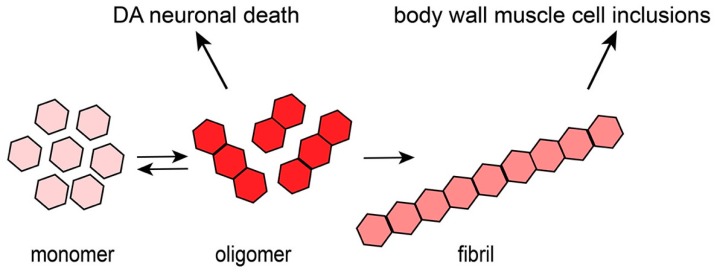
The process of α-syn oligomerization and fibrilization. Monomers of α-syn are small, soluble and have the capacity to associate together and oligomerize. These soluble oligomers are thought to be cytotoxic and act as the major contributor to neurodegeneration and neuronal deficits. Over time, oligomers have the potential to fold into β-sheet-containing fibrils, which constitute Lewy Bodies in PD.

A key pathological hallmark of PD is the presence of Lewy Bodies in DA neurons, which are primarily composed of an aggregation- and misfolding-prone protein called alpha-synuclein (α-syn), encoded by the human *SNCA* gene [10,11,12,13]. The significance of α-syn protein structure in PD is highlighted by specific amino acid substitutions found in rare familial cases. Notably, a change at residue 53, from an alanine to threonine (A53T), results in a form of the protein that exhibits a strong propensity toward rapid misfolding and oligomerization [14,15]. Another amino acid substitution, A30P, has also been shown to result in enhanced oligomerization of α-syn and is associated with early-onset PD [14,15]. Importantly, triplication of the wild-type *SNCA* locus has been found to be sufficient to cause PD in patients, thereby implicating an excess or accumulation of the *SNCA* gene product in PD pathology [16]. There is evidence suggesting that Lewy Bodies may actually be protective but this notion is still controversial [13,17,18,19]. However, it has been shown that the soluble oligomeric form of α-syn is the primary toxic species to DA neurons and not more mature polymeric forms that manifest as the fibrils found in Lewy Bodies [20,21] (Figure 1).

This review aims to illuminate and describe the utility and variety of *C. elegans* models available to study PD and bring to light some of the pivotal discoveries resulting from them. Although mutations in *PARK* genes and the addition of exogenous compounds and neurotoxins are used as the basis for many *C. elegans* models of PD, this review will concentrate on the models that revolve around the α-syn protein, given its central role in the disease. 

## 2. *C. elegans* Models of PD 

### 2.1. The First of Its Kind

There have been various models of PD generated in *C. elegans* that incorporate α-syn as a way to model the disease and each one provides advantages and disadvantages for researches investigating modulators of dopaminergic function and neurodegeneration (see Table 1 for a summary of these models). The first model established to examine α-syn in *C. elegans* was pioneered by Garry Wong and his research team in 2003 and utilized worms that overexpressed both wild-type and mutant (A53T) forms of α-syn, either in DA neurons only or pan-neuronally using the *dat-1* and *aex-3* promoters, respectively [22]. Since *C. elegans* is anatomically transparent, the morphology of the DA neurons is easily visualized in vivo utilizing Green Fluorescent Protein (GFP) as a co-injection marker and the extent of neurodegeneration was able to be established. These transgenic worms existed as stable lines and both α-syn and GFP were expressed as extrachromosomal arrays. This work showed that neuronal deficits including dopaminergic neurodegeneration could be induced through α-syn and that *C. elegans* could be used to model PD [22]. It is important to note however that this first model did not exhibit DA neuron defects that worsened with age, which is a key characteristic of the disease in humans. Young worms exhibited about the same levels of dopaminergic degeneration as older worms in this first model.

A set of transgenic nematodes constructed similarly to the previously described model, overexpressing wild-type, mutant A53T and mutant A30P α-syn under control of the *dat-1* promoter, restricting expression to the DA neurons, did not exhibit significant neurodegeneration compared to GFP only controls, even at 15 days of age [23]. Perplexingly, in a similar strain where YFP was expressed using the monoamine-specific promoter *cat-1*, YFP expression was significantly diminished in DA neurites in worms also overexpressing either wild-type or mutant forms of α-syn, implying that these structures were degenerated compared to controls [23]. This highlights the variability in the ability of similar α-syn models to produce phenotypes related to PD and could be due to the robustness of transgenic expression of α-syn which, in *C. elegans*, is a combined outcome of promoter strength and the variable copy number of a given expression construct in extrachromosomal transgenic arrays (as well as chromosomal integration sites for integrated transgenes). Nevertheless, dose-dependent α-syn neurotoxicity is a feature that correlates with the human etiology of PD and can be considered fortuitous for modeling. Notably, the mutant variants, but not the wild-type form, of α-syn induced an impairment of a DA-dependent mechanosensory behavior designated the basal slowing response (BSR). Therefore, this model is capable of recapitulating a key aspect of PD: loss of dopaminergic functionality [23].

Additionally, derivatives of this model that expressed α-syn pan-neuronally were utilized in an RNAi screen that identified genes involved in the endocytic pathway, including *apa-2*, *aps-2*, *eps-8* and *rab-7*, which modulated neuronal dysfunction due to α-syn cytotoxicity [24]. Although this α-syn model does not exhibit substantial progressive neurodegeneration of DA neurons as occurs in human PD, it has proven useful in identifying modulators of α-syn-related neuronal defects.

### 2.2. α-syn Aggregates in Muscle Cells

Taking advantage of the large size of body wall muscle cells in *C. elegans*, another PD model was created that allows researchers to visualize aggregation and inclusions of α-syn in vivo [25]. This model strain indeed showed that a fusion of the yellow GFP-variant, YFP, to human α-syn resulted in visible aggregates in the body wall muscle cells; these aggregates grew larger in size and abundance with age [25]. Additionally, these worms exhibited paralysis when α-syn aggregation reached a certain threshold of severity, providing a readout and the means to identify suppressors and enhancers of this aggregation [25]. Experiments using this model culminated in a genome-wide RNA interference (RNAi) screen aimed at discovering genes that modulate the formation of α-syn aggregates [25]. From this screen, 80 suppressors of α-syn::YFP aggregation (49 of which had human orthologs) were identified and it was determined that these newly identified genes were involved in cellular processes ranging from vesicular transport to lipid metabolism [25]. This α-syn model of PD, however, proved inconsistent with results obtained from a large whole-exome sequencing (WES) study in humans which identified many potential candidate modifiers of PD [30]. Importantly, many of these candidates were found to modulate PD-related phenotypes in a *Drosophila* retina-based model of α-syn-related neurodegeneration but none of these exhibited an effect in this *C. elegans* α-syn muscle-based model [30]. These inconsistencies in this *C. elegans* model could be due to the resulting α-syn::YFP aggregates being too mature, producing more inclusions which are associated with less toxic fibrils rather than the more toxic and detrimental oligomeric species or, possibly, due to the tissue-specific differences of α-syn expression.

### 2.3. Modeling Progressive Dopaminergic Neurodegeneration

**Figure 2:**
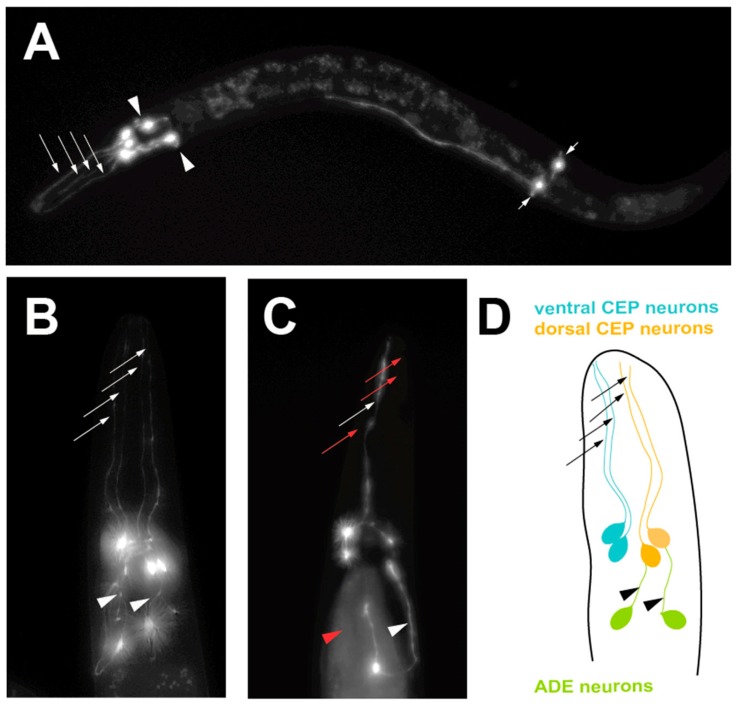
A progressive, age-dependent *C. elegans* α-syn model of PD [18]. (**A**) A worm expressing solely Green Fluorescent Protein (GFP) in the 8 DA neurons, with GFP being driven under the dopamine neuron-specific *dat-1* promoter for visualization. Arrows with long tails indicate the 4 CEP neuron processes, arrowheads indicate the 2 ADE neuron processes and arrows with short tails indicate the 2 PDE neuron processes. (**B**) A worm expressing both GFP and human, wild-type α-syn in the DA neurons being driven under the dopamine neuron-specific *dat-1* promoter, allowing visualization of the 6 DA neurons located in the head region. Arrows indicate non-degenerated CEP neuron processes and arrowheads indicate non-degenerated ADE neuron processes. (**C**) A worm expressing both GFP and human, wild-type α-syn in the DA neurons being driven under the *dat-1* promoter, allowing visualization of the 6 DA neurons located in the head region. Red arrows indicate degenerated CEP neuron processes and the red arrowhead represents a degenerated ADE neuron process. (**D**) A representation of the neuroanatomy of the 6 DA neurons in the head region of *C. elegans*. Ventral and dorsal CEP neurons are shown, along with the ADE neurons.

**Figure 3:**
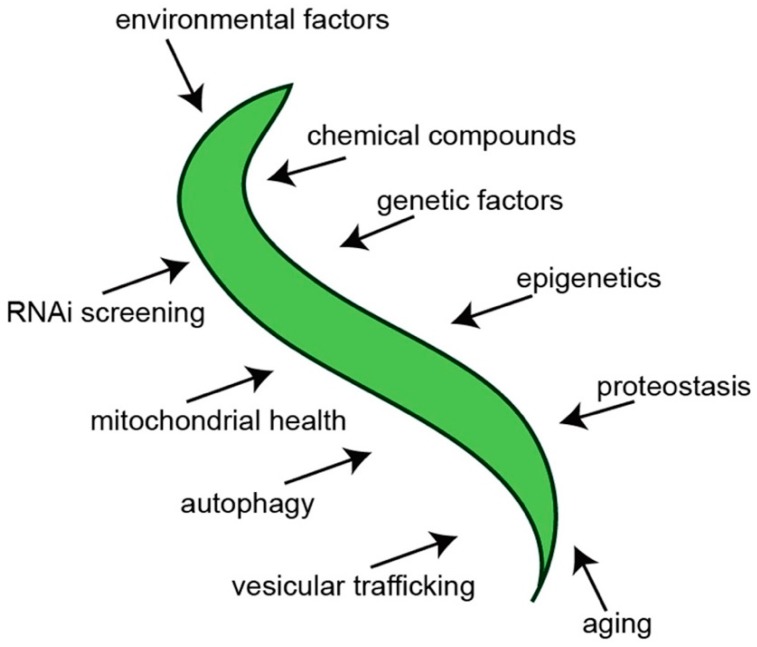
A summary of some of the putative factors and processes that can be investigated using *C. elegans* α-syn models of PD.

In an attempt to establish a more predictive model of PD in *C. elegans*, transgenic nematodes were generated that targeted multicopy expression of α-syn exclusively to the dopaminergic neurons [26]. Notably, these animals not only exhibited dopaminergic neurodegeneration but also displayed degeneration that was progressive and worsened with age (Figure 2). This phenotype is more analogous to how PD originates and manifests in humans. In this model, α-syn and GFP are overexpressed as distinct transgenes in the DA neurons, thereby allowing the visualization of the morphology of the neurons as they progressively degenerate [26]. Since *C. elegans* is anatomically transparent, the neurons are easily seen using fluorescent microscopy. Moreover, by delimiting expression to only the dopaminergic system, neuron morphology at the single-neuron level is readily ascertained, thereby providing unprecedented accuracy in quantification of neurodegeneration. This specific worm model of PD has aided in the discovery and/or characterization of numerous genes, regulatory RNAs, environmental compounds, chemical compounds and drugs and other factors that influence the levels of progressive dopaminergic neurodegeneration in vivo (Figure 3). Once candidates—whether they are genes, chemical compounds or anything else—are identified in a model of PD, they can be validated using this model and the capacity of these candidates to modulate dopaminergic neuroprotection in the face of the stressor α-syn can be determined. One such example of this can be seen in the characterization of the chemical compound N-aryl benzimidazole (NAB) [31]. This compound was first shown to reverse the molecular defects (in vesicle trafficking, metal ion homeostasis, mitochondrial function and dynamics, etc.) that manifested as the result of α-syn expression in yeast cells in a large screen [31]. Strikingly, NAB significantly decreased the extent of DA neurodegeneration in *C. elegans* and produced similar protective effects when tested on rat primary neuronal cultures and human induced pluripotent stem cells differentiated from PD patients [31,32].

Another showcase of the capacity of this model to successfully predict genes and processes that are involved with PD phenotypes that translate to higher organisms comes with its role in the discovery that *rab-1*, encoding the Rab guanosine triphosphatase (GTPase), is involved in ER-Golgi vesicle trafficking and mediates α-syn toxicity and its associated defects [33]. A strain of yeast that exhibited cellular and molecular deficits due to α-syn overexpression found that *C. elegans rab-1* (Ypt1p in yeast) was among the strongest suppressors of α-syn toxicity [33]. Protection due to *rab-1* was not restricted to the viability of yeast cells, however, and was found to significantly protect DA neurons from degenerating in this *C. elegans* model of PD [33]. Complementary studies showed that *rab-1* was neuroprotective in the context of α-syn in *Drosophila* and mammalian (primary cultures of rat midbrain neurons) models, strengthening the case for the predictive capacity of this *C. elegans* model of PD [33]. Perhaps most significantly, this model has proved to have the ability to uncover genes and proteins that not only modulate the misfolding or clearance of misfolded α-syn but also impact the neurotoxicity of α-syn. In a separate study, the worm ortholog of the highly conserved glycolytic enzyme, glucose-6-phosphate isomerase (GPI) emerged as a candidate from a RNAi screen of over 700 candidate genes previously associated with the *daf-2* pathway and the metabolic control of aging in *C. elegans*. In searching for modifiers of α-syn-related misfolding, GPI was characterized to be neuroprotective using this model [34]. Concurrent experiments investigating GPI in *Drosophila* and mice revealed that this neuroprotection was conserved across species, once again highlighting the capability of this α-syn worm model of PD to yield outcomes that are predictive and translational to more complex organisms [34].

A protein involved in lysosomal trafficking, VPS-41, was found to modulate α-syn misfolding and/or clearance in worms. Furthermore, its ability to modulate DA neuron degeneration induced by α-syn overexpression was determined using this *C. elegans* model of PD [27]. The overexpression of the human *vps-41* gene not only prevented DA neuron loss in this model but it also decreased levels of α-syn in human neuroglioma cells [35]. In addition, two specific domains residing on the VPS-41 protein, the AP-3 and clathrin heavy-chain repeat domains, were shown to be required for neuroprotection [36]. Not only can VPS-41 protect dopamine neurons from succumbing to the stress of α-syn accumulation in vivo, it also protects glutamatergic neurons—the major neuron subtype affected in Alzheimer’s disease—from succumbing to the stress associated with amyloid beta (Aβ) neurotoxicity [37]. These collective data support the potential for the use of *vps-41* as a prospective candidate for gene therapy.

A transcription factor that initiates the mitochondrial unfolded protein response (UPR^MT^) in response to mitochondrial dysfunction, ATFS-1, enhances DA neurodegeneration and deficits in DA-dependent behaviors when its function is lost in *C. elegans*, providing evidence that the UPR^MT^ plays a crucial role in enabling DA neurons to survive [38]. Contrarily, this dopaminergic model of α-syn-associated neurodegeneration in *C. elegans* provides evidence that activation by overexpression of ATFS-1, resulting in increased transcriptional activity, induces neurodegenerative phenotypes and behavioral deficits but dopaminergic neuroprotection in the presence of multiple α-syn variants, suggesting that ATFS-1 activation and thus the UPR^MT^ has a hormetic effect—beneficial at low levels but detrimental at high levels [39].

When determining the utility of any disease model, one must be able to gauge its ability to illuminate potential processes and factors that could one day be exploited to develop therapeutic targets and treatments for the disease in humans. Therefore, the strength of an α-syn-based *C. elegans* model depends on its ability to yield results that translate to mammalian models of the disease. This model of PD in particular, utilizing transgenics to overexpress human wild-type α-syn in DA neurons, has provided an abundance of insight into the fundamental molecular underpinnings of the disease. More significantly, it has also been used for both the discovery and functional validation of various potentially druggable targets and neuroprotective compounds that help to ameliorate the dopaminergic degeneration and neuronal deficits that are associated with α-syn pathophysiology. As an illustration of this, key modifier genes and corresponding proteins such as calcineurin, *rab-3, rab-8, rab-10*, rho GTPases, ATP13A2, cathepsin D, glucocerebrosidase (GBA) and 14-3-3 proteins, were found to modulate dopaminergic neurodegeneration – all using this specific α-syn worm model, and, either simultaneously or subsequently, were demonstrated to effect neurodegeneration in mammalian models [40,41,42,43,44,45,46]. Additionally, compounds identified and/or validated using this specific α-syn model, such as NAB and tetrahydroquinolines, attenuated DA neurodegeneration and accentuate the prospect of these compounds and/or related pathways for potential drug development [31,47]. Given the proven translational potential of this α-syn-based model of PD, it is poised to aid in more rapidly advancing our understanding of the pathology of the disease.

### 2.4. An Alternate Model for Investigation of α-syn Aggregation in Vivo

**Figure 4:**
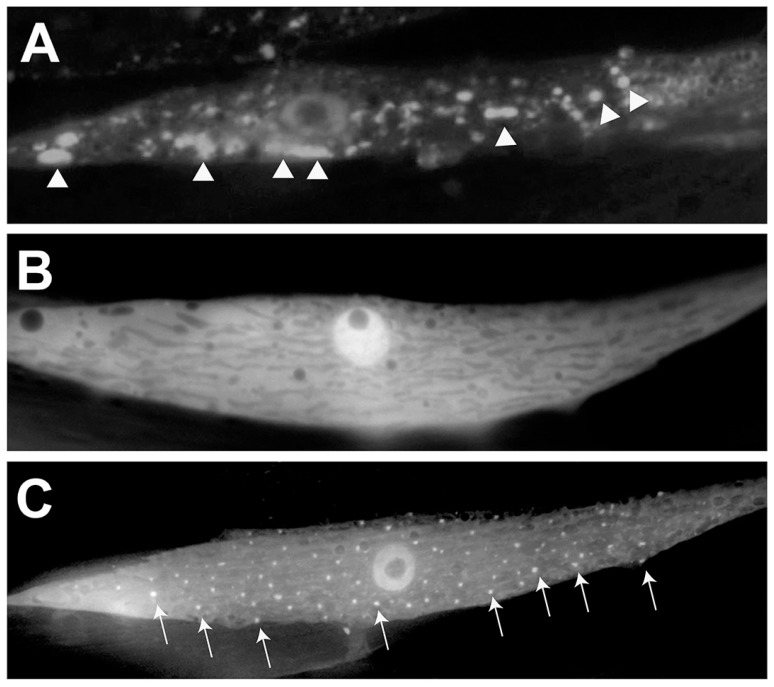
Use of *C. elegans* to model human α-syn misfolding. (**A**) A closeup image of a muscle cell in a transgenic worm expressing human, wild-type α-syn translationally fused to Green Fluorescent Protein (GFP) driven under the body wall muscle cell-specific *unc-54* promoter. Arrowheads indicate areas of large accumulations of misfolded α-syn. (**B**) A closeup of a worm muscle cell co-expressing human, wild-type α-syn translationally fused to GFP and the chaperone protein, TOR-2, driven as distinct expression constructs by the body wall muscle cell-specific *unc-54* promoter. TOR-2 serves to attenuate the amount of α-syn misfolding; the resulting GFP expression appears soluble and without puncta, indicating minimal misfolding/accumulation. (**C**) A closeup of a worm muscle cell expressing human, wild-type α-syn translationally fused to GFP in the presence of the chaperone protein TOR-2, both driven under the *unc-54* promoter. In this panel, a gene has been knocked down via RNAi that enhances α-syn misfolding. Arrows indicate areas of visible α-syn::GFP.

Another *C. elegans* model of PD overexpresses α-syn that is translationally fused with GFP in the body wall muscles of worms and exhibits an increase in the quantity and size of visible α-syn aggregates over time [27]. In order to discover genetic modifiers impacting the misfolding and accumulation of α-syn, the worm homolog of the human torsinA chaperone protein, TOR-2, was co-overexpressed with the α-syn-GFP fusion [27]. In this model, TOR-2 serves to significantly lessen the extent of misfolding in vivo so that the factors that are involved in the accumulation of aggregated α-syn can be more readily identified (as clearly depicted in Figure 4). The presence of TOR-2, for which its human homolog, torsinA, has been demonstrated to physically interact with α-syn [48], operationally serves to maintain a more oligomerized state, thereby facilitating identification of functional modifiers of toxicity in vivo. This α-syn/TOR-2 *C. elegans* model of PD was used in a large-scale RNAi screen searching for effectors of α-syn misfolding and/or clearance. This screen revealed that 125 candidate genes tested enhanced α-syn accumulation, which included orthologs of 5 established familial PD genes: *PARKIN/PARK2, DJ-1, PINK1, NURR1* and *PARK9* [27]. Importantly, beyond internal validation, this RNAi screen also identified numerous genes previously unassociated with α-syn or PD, including the aforementioned *vps-41* gene, revealing the power of this model to uncover novel factors with the potential for disease-modifying activity.

### 2.5. Single-Copy Transgenic Modeling of α-syn in C. elegans

A recently reported *C. elegans* PD model accomplishes a closer representation of conditions relating to the normal expression of α-syn in humans. This model ubiquitously expresses human α-syn as a single-copy transgene [28]. This is the scenario for the vast majority of humans that develop PD—levels of α-syn are the result of one haploid copy of the *SNCA* gene. The overt anatomical distinctions between worms and humans render aspects of this model less appealing. For example, the expression of α-syn is most abundant in the brain in humans, whereas this model expresses α-syn at the same levels in all tissues in worms. Nevertheless, this model shows some consistency with regards to PD phenotypes in humans—these worms exhibited defects in DA dependent behaviors such as thrashing and the basal slowing response and morphological defects in DA neurons such as dendritic blebbing [28]. However, a significant loss in DA neurons is not observed in α-syn worms compared to controls [28]. In spite of this, both DA dependent behaviors and DA neurodegeneration significantly worsen in these α-syn animals in conjunction with loss-of-function mutants in familial PD genes (*PARK2, PINK1, DJ-1, PARK9* homologs), thus emphasizing the synergistic interactions between these genes known to be involved in PD [28]. Interestingly, worm homologs of all these same recessive PD genes were also identified as modifiers of α-syn::GFP accumulation by RNAi screening using the previously described body-wall muscle model in which α-syn misfolding and aggregation was attenuated by chaperone co-expression [27]. Since this prior study employed a strain whereby α-syn was expressed as a multicopy transgene, it suggests that the single-copy nature of this newer model may not comparatively provide additional insights beyond those that have already been revealed when α-syn oligomerization is accounted for as a criterion in screening. Nevertheless, although this newer single-copy α-syn transgene model of PD does not produce progressive dopaminergic defects on its own, it provides another tool for researchers to examine potential PD modulating candidates in an invertebrate model that has levels of α-syn more akin to those in most people ultimately diagnosed with PD.

### 2.6. A Model to Explore the “Prion-Like” Nature of α-syn

In addition to the more mainstream hypothesis of DA neurons being more susceptible to the stress of α-syn due to their increased risk of generating reactive oxygen species and thus being exposed to more oxidative stress, there is also increasing evidence that misfolded α-syn itself is able to be transferred between cells, spreading from affected cell to unaffected cell [49,50,51]. A *C. elegans* model has been established that enables evaluation of this “prion-like” α-syn hypothesis through use of bimolecular fluorescence complementation (BiFC) to visualize neuron-to-neuron transfer of α-syn [29]. BiFC-induced GFP fluorescence is only observed when each complementary BiFC protein subunit comes into close proximity to one another. Ingeniously, each BiFC complement was fused to α-syn and driven under either the *ddr-2* or *tph-1* promoter [29]. The *ddr-2* promoter drives expression in neurons of the head and tail and in the ventral and dorsal nerve cords. The *tph-1* promoter drives expression exclusively in the 11 serotonergic neurons. These promoters and neuronal subtypes were selected for α-syn expression because they drive expression in distinct neurons that synapse to each other, enabling the opportunity to visualize spreading of α-syn in vivo. This model was deliberately designed in such a way that when fluorescence is observed, the implication is that α-syn has traveled from one neuron subtype to another and has dimerized. BiFC-induced fluorescence is seen in the expressing neurons as early as Day 1 and both the intensity and area of expression increases as the worms age [29]. This suggests that α-syn does indeed spread from neuron-to-neuron in this *C. elegans* model. When synaptic transmission was impaired due to mutations in genes involved in synaptic vesicle release, the spreading of α-syn was modulated, implying that this is the likely mode of exit and entry from neuron to neuron [29]. Interestingly, BiFC-induced fluorescence was decreased when genes involved in autophagy were knocked down via RNAi, suggesting that the process of autophagy is at least in part responsible for the clearance of α-syn and robustness of spreading [29]. Although this model provides insight into the spreading nature of α-syn, expression of α-syn is not in DA neurons. Thus, it is unclear if this spreading can specifically occur from DA neuron-to-DA neuron (or to another adjacent cell type) and detrimentally lead to dopaminergic neurodegeneration and neuronal deficits as reported in other α-syn-related *C. elegans* models of PD, as well as in humans.

## 3. Use of *C. elegans* to Discern the Role of Dopamine in α-syn-Induced Neurotoxicity

Indeed, the multicopy expression of the human α-syn protein in DA neurons is enough to induce dopaminergic neurodegeneration and/or neuronal deficits in many different animal models and in humans [22,23,24,25,26,27,28,52,53]. However, it has recently come to light that DA itself may play a crucial role in α-syn toxicity, adding to the complexity of the enigmatic mechanism through which α-syn promotes toxicity [54]. In a mouse model of PD in which a mutated version of α-syn implicated in familial PD (A53T) is overexpressed, increases in endogenous DA levels resulted in DA neuron loss and impaired locomotion compared to the wild-type version of α-syn [55]. Additionally, this increase in DA correlated with the increase of toxic oligomeric species of α-syn compared to controls [55]. Confirmation of this phenomenon came from *C. elegans*, in which it was shown that when A53T α-syn was mutated at an established site of interaction with DA, DA-induced toxicity and neurodegeneration was prevented, illuminating the role of α-syn-DA interaction in α-syn-induced toxicity [55]. Together, these observations highlight the importance of the interaction between DA and α-syn and give way to a new paradigm for α-syn-related PD pathology that accounts for the selective vulnerability of DA neurons in PD. These studies also set the stage for applying *C. elegans* to identify compounds that block this neurotoxic interaction. From a clinical standpoint, the fact that L-dopa has long been the primary medication prescribed to PD patients to alleviate symptoms of the disease, the intracellular consequences of excess DA levels may need to be a consideration, especially in individuals that may have a genetic predisposition toward increased levels of α-syn.

## 4. *C. elegans* as a Tool to Uncover Epigenetic Mechanisms of PD Pathology

It is becoming increasingly clear to the scientific community that epigenetics plays a major role in PD. Specific patterns of methylation and acetylation on histone tails dictate the expression levels of genes in that vicinity, potentially including genes related to PD. Differences in epigenetic landscapes between individuals can result from exposure to different environmental factors or from differences in the expression levels of regulatory RNAs. For example, double-stranded RNAs (dsRNAs) have the ability to produce secondary small RNAs which can be used by the secondary Argonaut protein HRDE-1 in *C. elegans* (involved in the RNAi pathway) to guide and deposit trimethylation marks on histone H3 on lysine residue 9 (H3K9) at genes of matching sequence [56,57,58]. This means that dsRNAs, like endogenous microRNAs (miRNAs) for instance, can dramatically and indirectly cause gene expression differences between individuals that may modulate susceptibility and/or resiliency to the neurotoxic effects of α-syn in the context of PD.

*C. elegans* affords researchers interested in investigating the role of epigenetics and dsRNAs in PD an ideal opportunity to do so. Due to its extremely short generation time, ease of propagation and fecundity, *C. elegans* is poised to lead the way in discovery of novel genes, regulatory RNAs and environmental factors that influence chromatin landscapes in ways that influence PD phenotypes. This is in part because epigenetic marks are known to be inherited from generation to generation and therefore worm models of PD are excellent for determining whether an effect resulting from an environmental factor, drug or exposure to a regulatory RNA can last for multiple generations after exposure has ceased. One study utilized *C. elegans* to uncover genes that increased and decreased the sensitivity of the histone deacetylase inhibitor (HDACi), valproic acid (VA), using a large RNAi synthetic lethality screen [59]. VA has been used to treat epilepsy, bipolar disorder and cancer and works to change the epigenetic landscape by removing acetyl groups on histone tails, thereby allowing for an increase in transcription in areas of the genome that are affected by VA. Since VA has been shown to dramatically protect DA neurons from degeneration in an α-syn PD model of *C. elegans*, differences in the state of chromatin may, in part, determine if neurons will be more susceptible or resilient to α-syn toxicity [60]. Methods like chromatin immunoprecipitation (ChIP) and ChIPseq in conjunction with these worm PD models have the potential to bring new insights with respect to how epigenetics may be playing a crucial role in both susceptibility and resilience to PD in a transgenerational context. Evidence for changes in DA neurodegeneration in subsequent generations after exposure to an initial modifier could provide a whole new perspective on the disease. As our understanding of epigenetics and the factors that influence differences between individuals and populations increases, it is becoming apparent that novel levels of regulation of the processes that are related to and influence the onset and progression of PD are at play.

## 5. Discussion

The variety of *C. elegans* models of PD that incorporate the transgenic expression of α-syn in distinct modalities exhibit specific and unique advantages and disadvantages for investigating PD on multiple fronts (Table 1). There is abundant evidence that worm models of PD can not only recapitulate clinical hallmarks of the disease that are found in patients (including DA neurodegeneration, a reduction in DA levels and function, as well as defects in behaviors related to movement and more) but also have the capability to uncover novel regulators of DA neurodegeneration not readily ascertained using other model organisms or methods. The ultimate goal of research related to PD is to exploit knowledge gained about the pathology of the disease and to use it to target processes that can inhibit, ameliorate, and/or reverse the damage inflicted on the nervous system. The most significant role for *C. elegans* as a model system for investigating PD is that this translational path can be accelerated. This nematode may be simple in form and function but most of the fundamental aspects needed to model the disease are present and a plethora of other experimental resources are readily available to advance hypotheses toward revelations. As the only metazoan animal on our planet for which a complete cell lineage has been determined and, a defined neuronal connectivity notwithstanding, this microscopic roundworm allows for the testing of large quantities of individuals and populations in a short amount of time in a cost-effective and statistically robust manner.

All the different models described in this review have served to increase our understanding of PD in the context of α-syn, which is arguably the factor most implicated in the initiation, progression and maintenance of the disease. Although treatments and cures may seem far off, these α-syn-related *C. elegans* systems are poised for the discovery of novel factors with the potential to reduce the toxicity of α-syn in DA neurons. Most importantly, *C. elegans* models have faithfully and reproducibly shown, time and time again, that results obtained in these systems are predictive for what may also be seen in mammals, including humans. The potential for *C. elegans* to yield insights into the epigenetic basis of PD, which may account for a much greater share of pathology than we currently realize, has never been greater. In this context of α-syn-associated neurodegeneration, use of the humble worm as a pre-clinical model for PD with demonstrated value in attaining translational outcomes is only likely to increase, especially when factoring in its utility for examining the intersection of gene-by-environmental components of PD etiology [61]. Through the amazing grace of this elegant little animal, science may have found a way to eventually save far more than a “wretch (worm?) like me.”

## Figures and Tables

**Table 1:** This table provides a summary of the advantages, disadvantages and genotypes of the strains used for each α-syn-related *C. elegans* model of Parkinson’s Disease (PD) detailed in this review. The published article in which each strain was created and first used is referenced below the description of the model.

Description of Transgenic *C. elegans* α-syn Model(s)	Advantages	Disadvantages	Genotype of Model Strain
Non-progressive neurodegeneration, α-syn expressed both pan-neuronally and exclusively in DA neurons [22]	Exhibits dopaminergic neurodegeneration and deficits in locomotion	Dopaminergic neurodegeneration and deficits in locomotion do not worsen with age; Limited reports of experimental use in the literature	Wild-Type α-syn or A53T α-syn mutation DA Neuron Specific ○ P*_dat-1_*::GFP; P*_dat-1_*::α-syn Pan-neuronal ○ P*_aex-3_*::GFP; P*_aex-3_*::α-syn
Limited neurodegeneration, Wild-Type and rare mutant forms of α-syn expressed either pan-neuronally or exclusively in DA neurons from different promoters [23,24]	Exhibits deficits in DA dependent behaviors Enables identication of modulators of α-syn neuronal toxicity	Dopaminergic neurodegeneration limited to neurites, not cell bodies and mainly observed with rare mutant forms of α-syn	Wild-type, A53T and A30P mutant forms of α-syn P*_dat-1_*:: α-syn, P*_ges-1_*::RFP P*_cat-1_*::EYFP, pRF4 *(rol-6)* P*_unc-51_*:: α-syn, *eri-1(mg366)*
α-syn misfolding in body-wall muscle cells [25]	Exhibits visible inclusions of α-syn that increase with age; expression in large muscle cells facilitates biochemical analysis and fluorescent protein interaction studies	Employs an α-syn fusion protein with YFP and, therefore, may not represent native structural dynamics; Identified modulators of α-syn misfolding in this model have proven inconsistent with *Drosophila* and mammalian models of PD	NL5901 (*pkIs2386* [P*_unc54_*::α-syn::YFP (pRP2386)*unc-119*(+)])
Progressive neurodegeneration with Wild-Type α-syn expressed in DA neurons [26]	Exhibits dopaminergic neurodegeneration that worsens with age; established utility in discerning translational outcomes	α-syn is overexpressed; expression limited to the 8 DA neurons, thereby complicating biochemical analysis and precluding evaluation of non-cell autonomous effects of α-syn on DA neurodegeneration	UA44 (*baIn11* [P*_dat-1_*:: α-syn, P*_dat-1_*::GFP])
α-syn in body wall muscle cells in a background where misfolding is attenuated by chaperone co-expression [27]	Presence of chaperone (TOR-2) maintains α-syn in a mono- to-oligomeric state Easy to identify both enhancers and suppressors of misfolding Results obtained in screens translate to *Drosophila* and mammals	α-syn is overexpressed as a fusion protein with GFP and thereby may not fully represent native structural dynamics; Presence of chaperone (TOR-2) in the strain background may complicate interpretation of α-syn-independent effects	Without TOR-2 co-expression: UA49 (*baIn2* [P*_unc-54_*::α-syn::GFP, *rol-6 (su1006)*]) With TOR-2 co-expression: UA50 (*baInl13* [P*_unc-54_*::α-syn::GFP, P*_unc-54_*::*tor-2*], *rol-6 (su1006*)])
Single-copy transgene of α-syn expressed ubiquitously [28]	Expression of α-syn is moderate Produces neuronal deficits in conjunction with *PARK* mutations	Does not exhibit dopaminergic degeneration or neuronal deficits on its own	JVR339 (*jerIs004* [P*_eft-3_*::α-syn: TagRFP: *let-858*_*unc-119*(+)]; *unc-119*(−); *vtIs7* [P*_dat-1_*::GFP(pRB490)])
“prion-like” α-syn spreading model [29]	Suitable model to investigate potential to lessen or worsen neuron-to-neuron α-syn transfer	Not clear if it exhibits dopaminergic neurodegeneration or neuronal deficits α-syn is not actually expressed in DA neurons specifically	JVR406 [(P*_ddr-2_*::BiFC1 (EGFH1-LINK-SYN); P*_tph-1_*::BIFC2 (SYN-EGFH2); *rol-6(su1006)*]
